# Correlation between serum cystatin C level and renal microvascular perfusion assessed by contrast-enhanced ultrasound in patients with diabetic kidney disease

**DOI:** 10.1080/0886022X.2022.2134026

**Published:** 2022-10-17

**Authors:** Ping Zhao, Nan Li, Lin Lin, Qiuyang Li, Yiru Wang, Yukun Luo

**Affiliations:** a School of Medicine, Nankai University, Tianjin, P. R. China; b Department of Ultrasound, First Medical Center, Chinese PLA General Hospital, Beijing, P. R. China; c State Key Laboratory of Kidney Diseases, National Clinical Research Center of Kidney Diseases, Beijing, P. R. China

**Keywords:** Ultrasound, contrast-enhanced ultrasound, diabetic kidney disease, cystatin C

## Abstract

**Objectives:**

To investigate the relationship between serum cystatin C (CysC) levels and renal microvascular perfusion in patients with diabetic kidney disease (DKD).

**Methods:**

A total of 57 patients with high CysC levels and 45 patients with normal CysC levels were enrolled. Data on clinical characteristics and laboratory examination results were also collected. Contrast-enhanced ultrasound (CEUS) of the kidneys was successively performed. The time-intensity curve (TIC) and related quantitative parameters of the kidneys were obtained by CEUS and the correlations between CysC and CEUS parameters were analyzed.

**Results:**

Compared to the normal CysC group, the high CysC group had significantly lower wash-in area under the curve (WiAUC), wash-out area under the curve (WoAUC), and wash-in and wash-out area under the curve (WiWoAUC). In the normal CysC group, patients with Stage III chronic kidney disease (CKD) had higher AUCs than those with Stage I–II CKD (*p* < 0.05). In the high CysC group, patients with Stage IV–V CKD had lower wash-in AUC compared to patients with Stage I–II CKD (*p* = 0.023). The renal cortex microvascular perfusion parameters AUCs were positively correlated with the estimated glomerular filtration rate (GFR) (*r* = 0.280, 0.222, and 0.243), and CysC was inversely correlated with AUCs (*r*= −0.299, −0.251, and −0.273).

**Conclusions:**

CEUS parameters reflected changes in renal microvascular perfusion in patients with DKD, while AUCs might be useful indicators of declining GFR in DKD patients with increased CysC.

## Introduction

DM has become one of the most serious health problems worldwide because of its high prevalence, disability, and mortality rates. Microvascular and macrovascular complications associated with DM are also increasing sharply [1]. Diabetic kidney disease (DKD) is one of the most common microvascular complications associated with DM [2]. Cohort studies reported that 20–40% of diabetes cases may develop DKD [3,4]. DKD is the primary cause of chronic kidney disease (CKD) and an important risk factor for cardiovascular disease and premature death in patients with diabetes [5,6]. Early DKD diagnosis can inform clinical treatment decisions and delay DKD progression.

At present, the formula calculation of serum creatinine (Scr) level is the most used clinical method to estimate the glomerular filtration rate (GFR). However, Scr cannot sensitively reflect GFR in early renal impairment because Scr levels increase only when GFR had decreased to 50% of the normal value [7–9]. Cystatin C (CysC) has recently been proposed as an ideal endogenous marker for evaluating glomerular filtration function [10]. CysC concentration is not believed to be related to sex, age, or muscle content [11]. In addition, recent studies have shown that serum CysC participates in the pathological processes of vascular remodeling and neovascularization, which are closely related to the occurrence and development of diabetic microangiopathy [12].

As a noninvasive and sensitive blood pool imaging method, contrast-enhanced ultrasound (CEUS) can sensitively evaluate the blood perfusion of organ microcirculation and provide microcirculation information for clinical diagnosis [13]. Moreover, the contrast agent used in CEUS, gas microbubbles, has no nephrotoxicity due to their metabolic pathway [14]. Previous studies have confirmed that the quantitative parameters of CEUS can be used for the early diagnosis of DKD [15] and to evaluate disease progression or prognosis [16,17].

Based on the results described above, the correlations between CysC and CEUS parameters are of interest. Whether CysC is directly involved in renal microvascular injury remains unclear. Therefore, this study investigated the relationship between CysC and quantitative parameters of CEUS in patients with DKD.

## Materials and methods

### Study design and patients

The participants were recruited from our hospital. All patients underwent renal biopsies to confirm kidney injury. The inclusion criteria were type 2 DM patients with kidney injury based on the renal biopsies results. The exclusion criteria were non-CKD; allergy to the contrast-enhanced agent; severe heart, brain, or pulmonary disease; pregnancy; congenital renal abnormalities (such as duplicate kidneys and fusion kidneys) or renal cysts or renal tumors; unilateral kidney atrophy; a combination with unilateral or bilateral renal artery stenosis; complications, such as systemic disease or urinary tract infection; and age < 18 or > 80 years. The clinical data collected included age, sex, body mass index (BMI), systolic blood pressure (SBP), and diastolic blood pressure (DBP). Before CEUS examination, blood was drawn from all subjects for blood urea nitrogen (BUN), Scr, and serum CysC measurement. According to the biochemical test results, the normal range of CysC levels was 0.45–1.25 mg/L, with levels >1.25 mg/L defined as high CysC. The patients were then divided into the normal and high CysC groups. The estimated glomerular filtration rate (eGFR) was calculated by the CKD-EPI formula: Scr ≤0.9 mL/dL:eGFR = 144 × (Scr/0.9) − 0.411 × (0.993)*Age and Scr >0.9 mL/dL: eGFR = 144 × (Scr/0.9) − 1.209 × (0.993)*Age for male; Scr ≤0.7 mL/dL:eGFR = 144 × (Scr/0.7) − 0.329 × (0.993)*Age and Scr > 0.7 mL/dL:eGFR = 144 × (Scr/0.7) − 1.209 × (0.993)*Age for female). Patients in each group were stratified according to eGFR level: patients with Stage I–II CKD (≥60 mL/min/1.73 m^2^), patients with stage III CKD (60 > eGFR ≥30 mL/min/1.73 m^2^), and patients with Stage IV–V CKD (<30 mL/min/1.73 m^2^). This study was approved by the hospital ethics committee (approval number S2017-152-02). All participants signed an informed consent form for ultrasound imaging before the examination.

### CEUS

The ultrasonographic device used in this study was an Acuson S2000 system equipped with a C6-2 transducer (Siemens, Erlangen, Germany), with a frequency of 2–6 MHz. The patients lay on their side. The left kidney was displayed to capture its form, echo, and size in the long-axis view. The contrast agent SonoVue (Bracco, Milan, Italy), consisting of sulfur hexafluoride microbubbles, was prepared according to the manufacturer’s instructions. After the CEUS mode was initiated, 0.5 mL of contrast agent was administered through the superficial elbow vein using the bolus injection method, followed by a 5 mL flush with 0.9% saline. Image acquisition was performed by a physician with more than 5 years of CEUS experience. The patients were asked to hold their breath for at least 30 s and then breathe slowly and smoothly for up to 3 min.

### Image analysis

**Figure 1.**
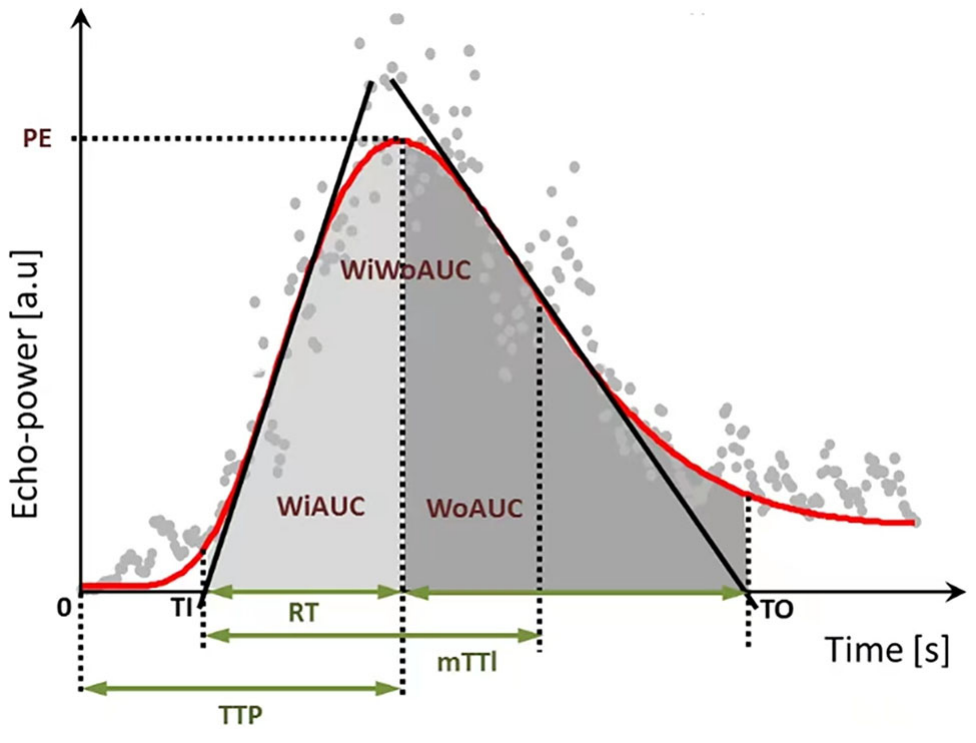
A representative diagram of a curve fitting diagram of TIC. PE: peak enhancement; WiAUC: wash-in area under the curve; RT: rise time; mTTI: mean transit time local; TTP: time to peak; WoAUC: wash-out area under the curve; WiWoAUC: wash-in and wash-out area under the curve [18].

The ultrasound sequences were output into digital imaging and communication in medicine (DICOM) format. Renal perfusion was analyzed in VueBox version 7.0 (Bracco SpA, Milan, Italy). Three regions of interest (ROIs) were defined: a reference ROI and two analysis ROIs. The segmental or interlobular arteries were set as the reference ROI in each kidney image and two ROI regions were set at the cortical zones in each image. The analysis ROI was placed on the middle pole to the lower pole of the renal cortex. The area of the ROI was 0.3 cm^2^. Movement compensation was applied to all participants. The DICOM data of the ROI were converted into echo-power data. A time-intensity curve (TIC) of the ROI was generated, from which the analysis parameters were calculated (Table 1, Figure 1). The parameters included ratio of the peak enhancement to the reference ROI (PE%); wash-in area under the curve (WiAUC, a.u.); rise time (RT, s); mean transit time (mTTI, s); time to peak (TTP, s); wash-out AUC (WoAUC, a.u.); wash-in and wash-out area under the curve (WiWoAUC, a.u.). The values obtained in the two ROIs from the leftrenal cortical zones were averaged for the analysis. To guarantee the quality of the data analysis, the goodness of fit (GOF) in all analyses was not <75%.

**Table 1.** Time-intensity curve parameters in the CEUS analysis.

Parameter	Definition
PE, %	Ratio of the peak enhancement to the reference ROI
WiAUC, a.u	Wash-in area under the ascending curve
RT, s	Rise time, independent of the time of origin
mTTI, s	mean transit time, local
TTP, s	Time to peak
WoAUC, a.u	Wash-out area under the descending curve
WiWoAUC, a.u	WiAUC + WoAUC
QOF, %	Quality of fit between the echo power signal and f(*t*)

The unit a.u. indicates arbitrary unit, f(*t*) is the instantaneous signal of the time(*t*).

### Statistical analysis

Continuous variables with normal distributions were expressed as means ± SD; otherwise, they were expressed as medians (IRQ). Categorical data were expressed as numbers (proportions). Comparisons between groups were performed using Student’s *t* tests, while those among three groups were performed using one-way analysis of variance or Mann–Whitney *U* tests, according to the type of data. Differences in sex distribution between the groups were analyzed using chi-squared tests. Spearman’s correlation analysis was performed to assess the relevance of CysC and renal microvascular perfusion parameters. All statistical analyses were conducted using IBM SPSS Statistics version 26.0 (IBM Corp., Armonk, NY). *p* < 0.05 was considered statistically significant.

## Results

### General characteristics

**Figure 2.**
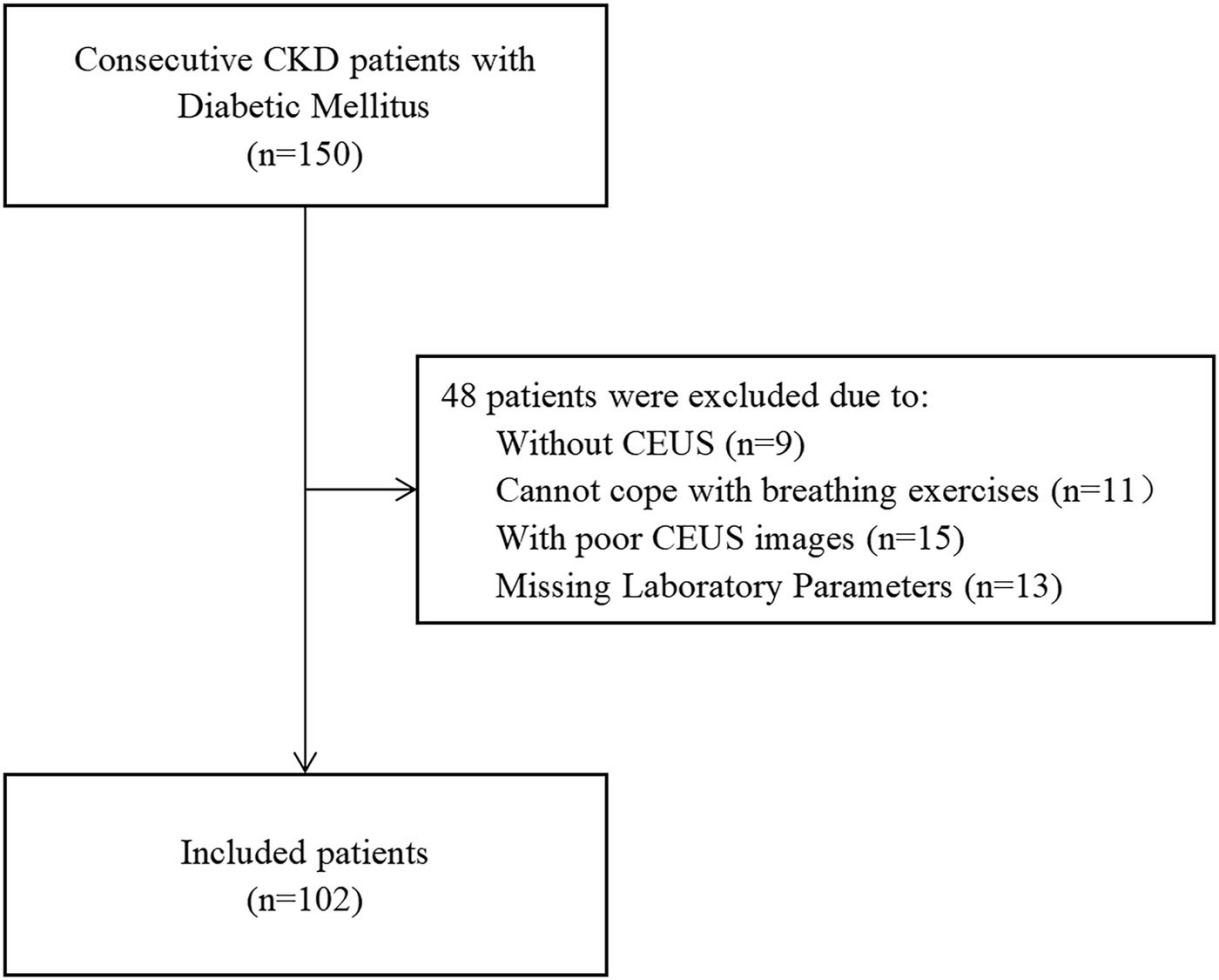
Study population chart. CKD: chronic kidney disease; CEUS: contrast-enhanced ultrasound.

Among 150 recruited patients with DM and CKD, 48 were excluded (Figure 2). Thus, 102 patients were included in the study. Among them, 45 patients and 57 patients had normal and high CysC levels, respectively. The mean age of the patients with normal CysC levels was comparable to that of patients with high CysC (50.07 ± 14.21 vs. 50.05 ± 12.23 years; *p=*0.996; Table 2). The distribution of sex, BMI, DBP, kidney long diameter, thick diameter, and wide diameter did not differ significantly between the groups (*p* > 0.05, Table 2). Compared to the normal CysC group, the high CysC group had significantly higher SBP, BUN, Scr, and CysC levels and lower eGFR and kidney cortical thickness (*p* < 0.01, Table 2).

**Table 2.** Clinical and ultrasound parameters between normal Cys C and high Cys C groups.

	Normal Cys C	High Cys C	*p*
*N*	45	57	
Age (year)	50.07 ± 14.21	50.05 ± 12.23	0.996
Sex (%male)	64.4	71.9	0.419
BMI (kg/m^2^)	25.81 ± 3.90	26.53 ± 3.36	0.319
SBP (mmHg)	132.51 ± 24.5	148.72 ± 23.19	0.001
DBP (mmHg)	81.53 ± 15.06	85.49 ± 14.00	0.173
eGFR (mL/min/1.73 m^2^)	90.55 (67.14–100.61)	32.00 (24.3–49.12)	<0.001
BUN (mmol/L)	5.15 (4.42–6.32)	10.50 (7.80–12.89)	<0.001
Scr (umol/L)	76.2 (63.55–95.85)	165.20 (128.10–217.30)	<0.001
Cys C (mg/L)	1.01 (0.86–1.14)	2.23 (1.68–2.72)	<0.001
Kidney long diameter (cm)	11.4 ± 1.06	11.09 ± 0.84	0.098
kidney thick diameter (cm)	4.96 ± 0.50	4.95 ± 0.49	0.906
Kidney wide diameter (cm)	5.3 ± 0.61	5.30 ± 0.64	0.721
Kidney cortical thickness (cm)	0.89 ± 0.10	0.81 ± 0.10	<0.001
PE [%]	32.28 (16.96–54.75)	26.22 (14.59–43.45)	0.251
RT [s]	5.78 (4.24–9.29)	6.12 (5.07–9.94)	0.214
mTTI [s]	66.10 (43.69–88.58)	71.39 (48.39–98.33)	0.292
TTP [s]	9.98 (6.78–14.06)	8.70 (7.15–13.88)	0.78
WiAUC [a.u]	31,569.57 (19,605.86–58,783.78)	20,622.80 (13,185.04–34,519.56)	0.004
WoAUC [a.u]	75,879.25 (43,373.90–116,212.88)	52,428.06 (27,668.86–87,713.88)	0.026
WiWoAUC [a.u]	108,758.13 (59,298.50–166,927.94)	69,974.66 (40,055.89–130,082.81)	0.012

BMI: body mass index; SBP: systolic blood pressure; DBP: diastolic blood pressure; eGFR: estimated glomerular filtration rate; BUN: blood urea nitrogen; Scr: serum creatinine; Cys C: cystatin C.

### High CysC with lower renal perfusion in DKD

**Figure 3.**
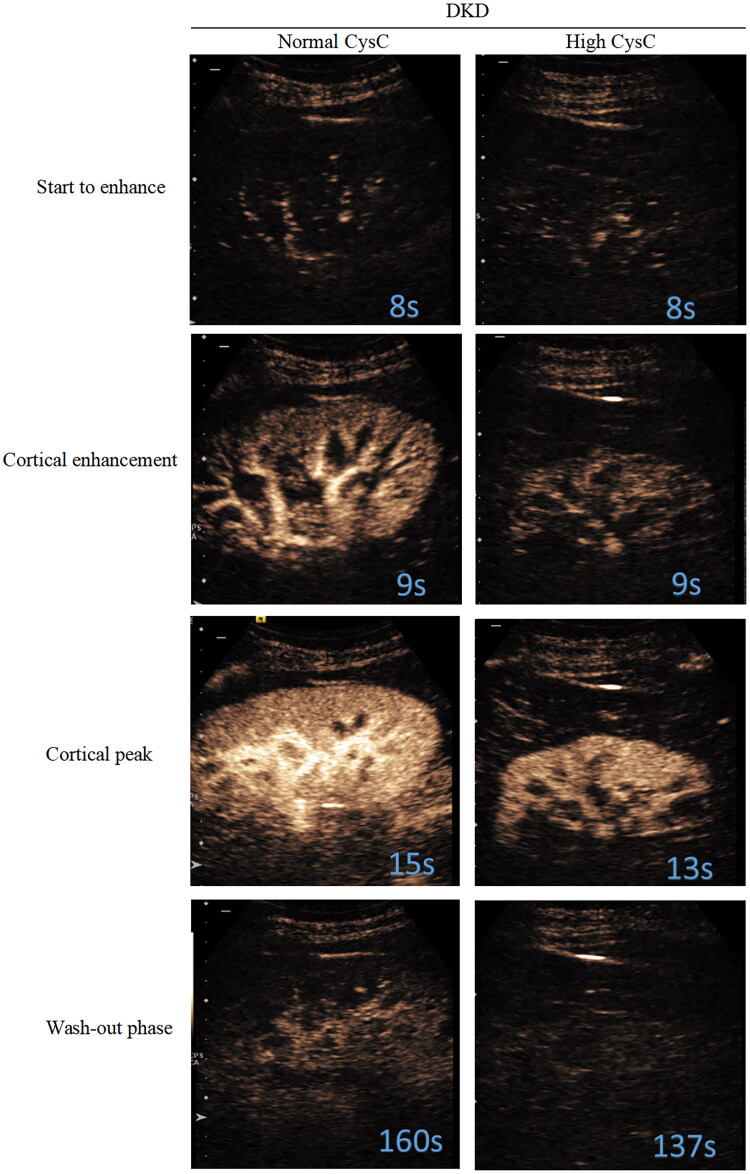
Representative serial contrast-enhancement images in groups. All subjects went through 4 stages including ‘start to enhance’, ‘cortical enhancement’, ‘cortical peak’, and ‘wash-out phase’. CysC: cystatin C; DKD: diabetic kidney disease.

All subjects were recorded at four stages: ‘start to enhance’, ‘cortical enhancement’, ‘cortical peak’, and ‘wash-out phase’ (Figure 3). Quantitative analysis of the CEUS parameters (Table 2) showed that, compared to the normal CysC group, the high Cys C group had significantly lower WiAUC, WoAUC, and WiWoAUC in the renal cortex (*p* < 0.05, Table 2). Other parameters, such as PE%, RT, mTTI, and TTP did not differ significantly between the groups.

### Subgroups of CKD stage in the normal and high CysC groups

The patients in the normal Cys C and high Cys C groups were divided into three subgroups based on CKD stage. In the normal CysC group, 39 patients had Stage I–II CKD, six patients had Stage III CKD, and no patients had Stage IV–V CKD. In the high CysC group, six patients had Stage I–II CKD, 26 patients had Stage III CKD III, and 25 patients had Stage IV–V CKD. The clinical characteristics of the participants according to CKD stages are shown in Table 3.

**Table 3.** Clinical and ultrasound parameters on three subgroups based on CKD stage in normal Cys C and high Cys C group.

	Normal Cys C				High Cys C			
	CKD I–II	CKD III	CKD IV–V	*p*	CKD I–II	CKD III	CKD IV–V	*p*
*N*	39	6	0		6	26	25	
Age (year)	48.77 ± 14.48	58.5 ± 9.29	NA	0.12	46.17 ± 14.25	50.85 ± 11.91	50.16 ± 12.45	0.706
SEX (%male)	61.5	83.3	NA	0.562	50	73.1	76	0.47
BMI (kg/m^2^)	25.86 ± 3.95	25.5 ± 3.90	NA	0.84	26.22 ± 3.18	26.39 ± 3.49	26.75 ± 3.39	0.905
SBP (mmHg)	131.28 ± 22.33	140.5 ± 37.48	NA	0.397	138.17 ± 27.29	140.73 ± 20.23	159.56 ± 21.31*^#^	0.006
DBP (mmHg)	81.49 ± 15.06	81.83 ± 16.45	NA	0.959	83.33 ± 15.57	81.15 ± 12.76	90.52 ± 13.77	0.051
eGFR (mL/min/1.73 m^2^]	94.33 (70.14–103.96)	52.44 (48.57–55.00)	NA	<0.001	75.35 (69.33–85.39)	40.95 (33.29–51.45)*	22.36 (15.90–25.86)*^#^	<0.001
BUN (mmol/L)	5.1 (4.29–6.01)	6.48 (4.87–10.83)	NA	0.086	6.88 (5.07–9.88)	8.12 (6.70–11.21)	12.61 (10.77–15.59)*^#^	<0.001
Scr (umol/L)	73.1 (61.9–85.5)	103.5 (96.08–116.38)	NA	<0.001	85.45 (55.16–114.63)	134.40 (123.08–153.95)	232 (191.8–335.75)*^#^	<0.001
Cys C (mg/L)	0.98 (0.79–1.11)	1.18 (1.06–1.22)	NA	0.009	1.43 (1.29–1.85)	1.77 (1.47–2.16)	2.6 (2.4–3.24)*^#^	<0.001
PE [%]	32.12 (15.18–51.12)	47.94 (23.65–66.49)	NA	0.256	27.07 (23.74–41.41)	30.84 (20.81–48.32)	16.01 (10.78–42.91)	0.169
RT [s]	5.50 (3.97–9.06)	8.26 (6.93–9.98)	NA	0.083	7.92 (4.44–13.79)	5.77 (5.14–9.93)	6.52 (5.01–10.43)	0.820
mTTI [s]	63.78 (43.74–88.56)	72.01 (34.58–102.76)	NA	0.894	65.69 (52.40–91.33)	76.64 (50.23–100.17)	67.14 (40.39–112.53)	0.955
TTP [s]	8.56 (6.66–12.26)	15.10 (10.00–15.83)	NA	0.062	11.27 (7.55–18.83)	8.90 (7.15–13.93)	8.39 (7.09–13.86)	0.692
WiAUC [a.u]	28,658.38 (19,112.15–48,340.66)	49,155.92 (41,912.05–117,541.05)	NA	0.021	46,793.72 (24,291.25–104,981.96)	23,960.64 (13,423.11–39,901.48)	16,334.55 (9274.36–27,314.12)*	0.023
WoAUC [a.u]	70,310.54 (40,230.60–103,892.65)	118,074.34 (85,285.03–234,296.98)	NA	0.030	112,646.24 (49,240.22–236,890.36)	53,169.23 (29,502.54–89,426.67)	45,234.16 (23,668.41–82,891.80)	0.101
WiWoAUC [a.u]	94,023.21 (58,902.17–163,778.71)	161,711.90 (135,474.63–351,838.03)	NA	0.028	159,439.96 (73,531.47–341,872.32)	75,666.86 (43,891.67–135,582.26)	68,350.99 (32,730.90–106,112.07)	0.076

* *p* < 0.05, compared to CKD I–II stage patients; ^#^*p* < 0.05, compared to CKD III stage patients.

BMI: body mass index; SBP: systolic blood pressure; DBP: diastolic blood pressure; eGFR: estimated glomerular filtration rate; BUN: blood urea nitrogen; Scr: serum creatinine; Cys C: cystatin C.

In the normal CysC group, patients with Stage I–II CKD had higher eGFR and lower Scr and CysC levels than those with stage III CKD (*p* < 0.01). In the high CysC group, patients with Stage IV–V CKD had higher SBP, BUN, Scr, and CysC levels and lower eGFR than patients with Stage I–II and III CKD (*p* < 0.01, Table 3).

### Renal perfusion in subgroups of CKD stage in the normal and high CysC groups

In the normal CysC group, patients with Stage III CKD had higher WiAUC, WoAUC, and WiWoAUC than those with Stage I–II CKD I (*p* < 0.05, Table 3). In the high CysC group, patients with Stage IV–V CKD had lower WiAUC compared to patients with Stage I–II CKD I (*p* = 0.023, Table 3).

### Correlations between renal microvascular perfusion and laboratory parameters

**Figure 4.**
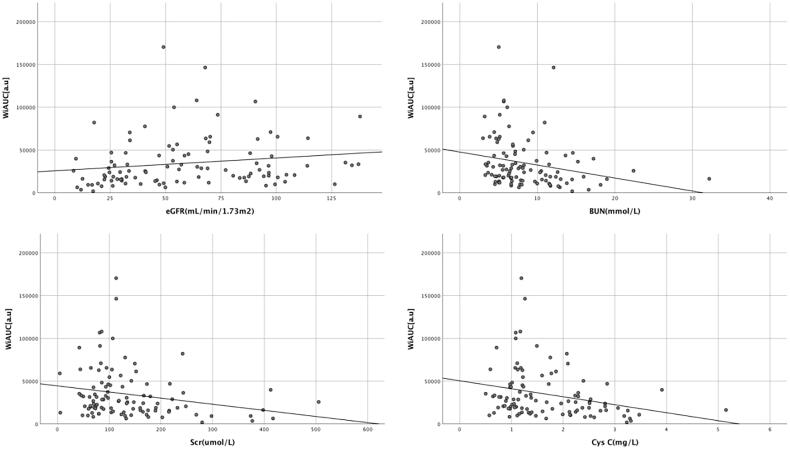
Correlation between renal cortex microvascular perfusion parameter WiAUC and laboratory parameters. WiAUC: wash-in area under the curve; eGFR: estimated glomerular filtration rate; BUN: blood urea nitrogen; Scr: serum creatinine; CysC: cystatin C.

**Figure 5.**
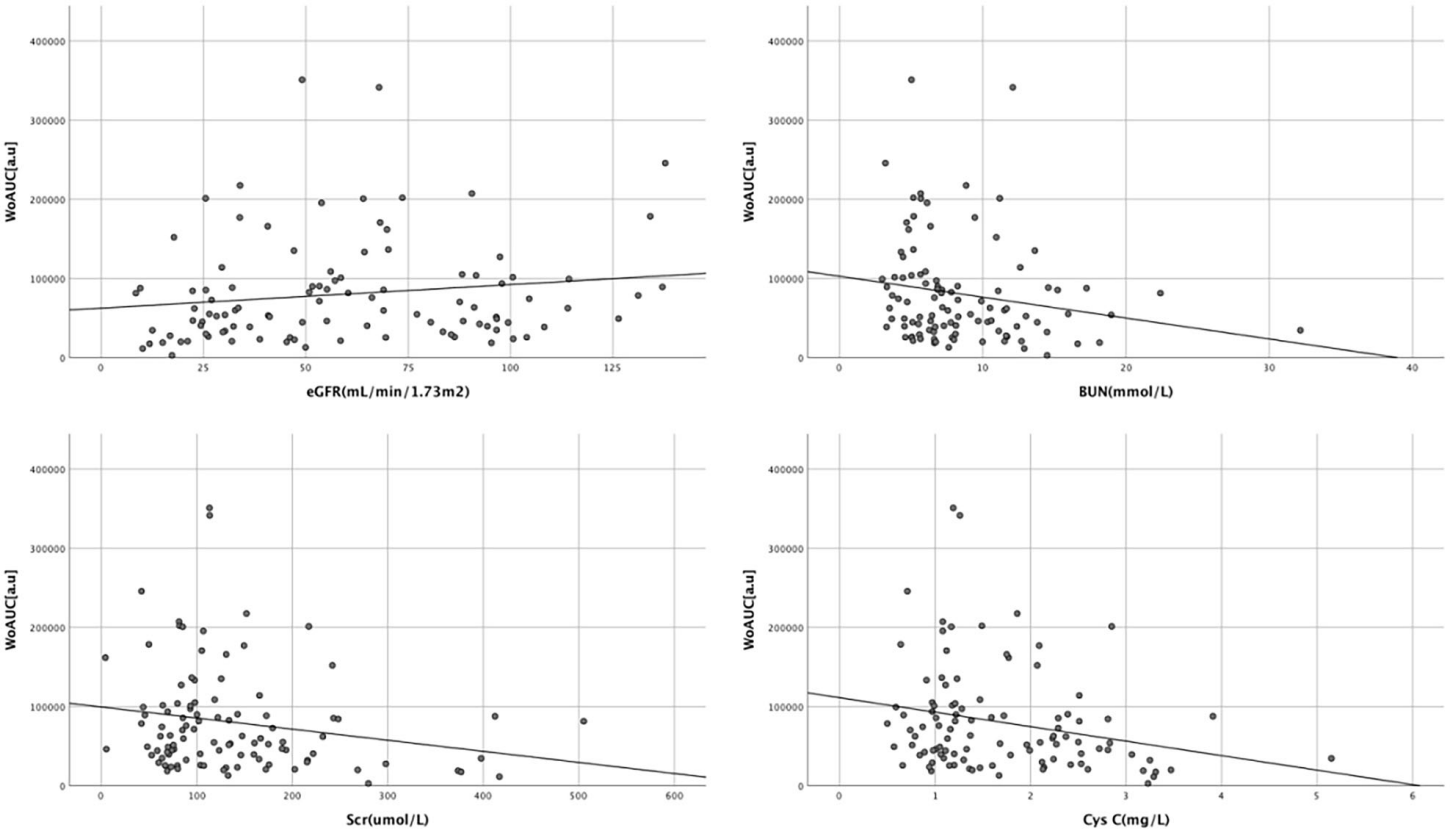
Correlation between renal cortex microvascular perfusion parameter WoAUC and laboratory parameters. WoAUC: wash-out area under the curve; eGFR: estimated glomerular filtration rate; BUN: blood urea nitrogen; Scr: serum creatinine; CysC: cystatin C.

**Figure 6.**
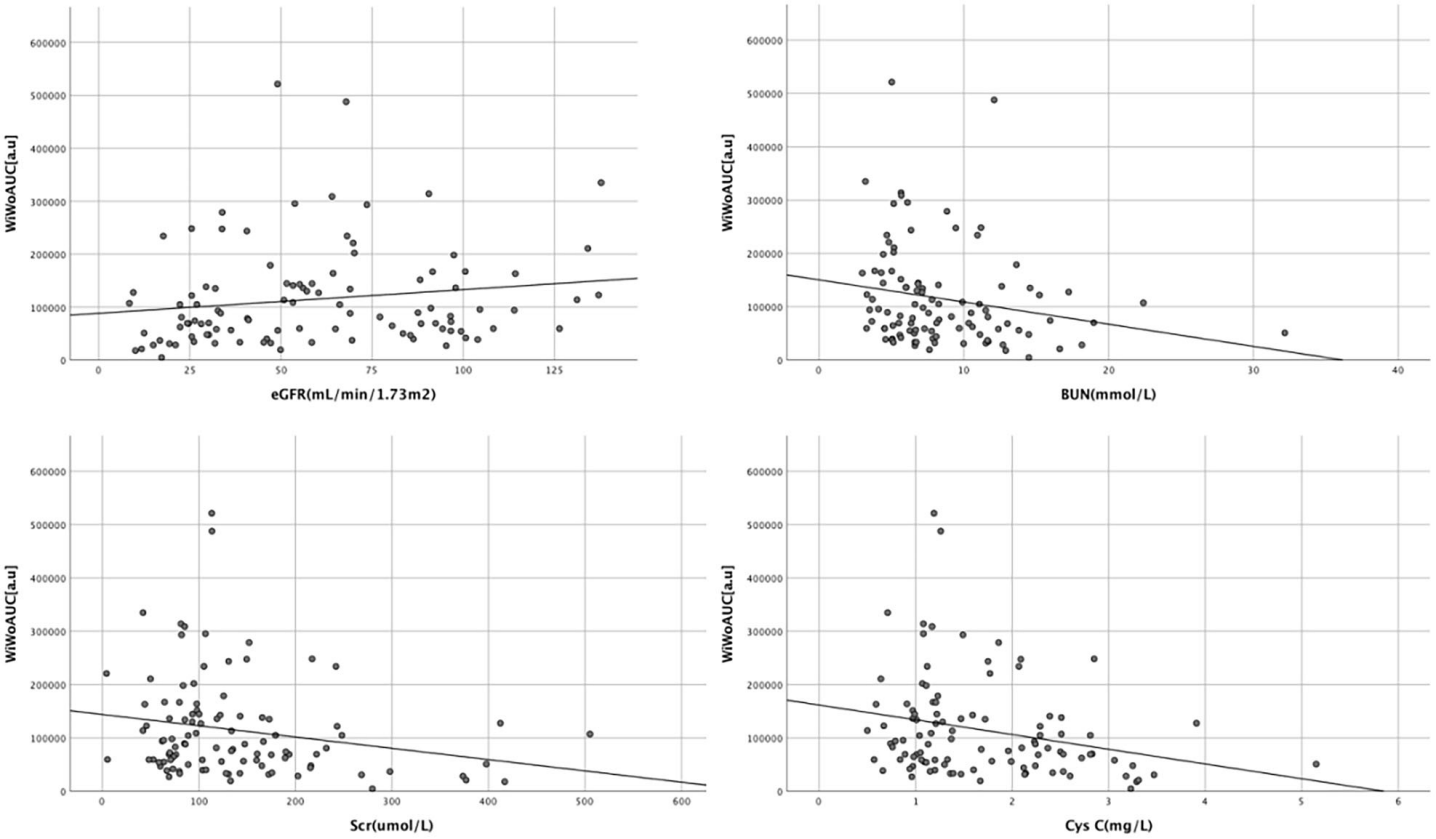
Correlation between renal cortex microvascular perfusion parameter WiWoAUC and laboratory parameters. WiWoAUC: wash-in and wash-out area under the curve; eGFR: estimated glomerular filtration rate; BUN: blood urea nitrogen; Scr: serum creatinine; CysC: cystatin C.

The renal cortex microvascular perfusion parameters WiAUC, WoAUC, and WiWoAUC were positively correlated with eGFR (Table 4). And WiAUC, WoAUC, and WiWoAUC were inversely correlated with CysC, BUN, and Scr (Table 4, Figures 4–6).

**Table 4.** Correlation between parameters of renal cortex microvascular perfusion and laboratory parameters.

	WiAUC		WoAUC		WiWoAUC	
	*r*	*p*	*r*	*p*	*r*	*p*
eGFR (mL/min/1.73 m^2^)	0.28	0.004	0.222	0.025	0.243	0.014
BUN (mmol/L)	−0.316	0.001	−0.245	0.013	−0.275	0.005
Scr (umol/L)	−0.241	0.015	−0.196	0.048	−0.215	0.03
Cys C (mg/L)	−0.299	0.002	−0.251	0.011	−0.273	0.006

eGFR: estimated glomerular filtration rate; BUN: blood urea nitrogen; Scr: serum creatinine; Cys C: cystatin C.

## Discussion

DKD onset is often not recognized and its clinical manifestations lack specificity. Albuminuria, Scr, and other laboratory indicators commonly used to evaluate renal function lack sensitivity for the early-stage evaluation and diagnosis of renal function damage [19]. However, CysC, which has a low molecular weight and positive charge, can freely pass through the negatively-charged basement membrane to enter the renal tubules through glomerular filtration [20]. CysC levels are not affected by inflammation and metabolic diseases; thus, they may better reflect renal function than Scr, especially in mild to moderate renal function damage [21]. This study focused on patient CysC levels. After dividing the patients into two groups according to CysC levels, we observed more severe renal injury in the high CysC group. The SBP, BUN, and Scr levels were higher, and the eGFR was lower in the high CysC group. These results are consistent with those reported previously [22].

This study also applied CEUS, an emerging tool for evaluating the microcirculation of the kidney in DKD. CEUS can quantitatively evaluate renal microcirculation perfusion and improve the sensitivity and specificity of ultrasound in the diagnosis of DKD. CEUS has been used to evaluate renal microvascular perfusion in CKD, acute kidney injury, and other kidney diseases [223–25]. The histological manifestations of DKD include glomerulosclerosis, renal interstitial fibrosis, renal tubule atrophy, decreased peri-tubular capillaries, and inflammatory reactions. These changes can lead to decreased renal blood perfusion [26]. In our study, we used a series of parameters to compare patients with normal and high CysC levels. Although PE, RT, mTTI, and TTP were not sensitive enough to show significant differences between the two groups, they showed a change trend in different levels of CysC. The WiAUC, WoAUC, and WiWoAUC were lower in the high CysC group than in the normal CysC group. The AUC-related parameters were related to the distribution volume, blood flow velocity, and perfusion time of the contrast medium. When the dose of the ultrasound instrument and contrast medium is constant, AUC is affected by blood flow velocity and blood distribution volume. Thus AUC shows a linear correlation with tissue blood flow [27]. Therefore, these can be used for the overall quantitative evaluations of the whole process of renal CEUS perfusion, are more sensitive than other parameters, and have higher reference values. Our findings were consistent with those of studies that suggested that alterations of CEUS in renal perfusion are key processes in CKD progression [28].

As CysC is superior to sCr-based CKD staging in evaluating renal function of early-stage CKD [22,29], this study was grouped based on the level of CysC, and found that ultrasound contrast parameters WiAUC, WoAUC, and WiWoAUC were able to evaluate renal blood perfusion in this part of patients. Then we focused on the associations of CKD stages with CysC levels. The results revealed patients with Stage I–II CKD in the high CysC group and with Stage III CKD in the normal CysC group. However, in the normal CysC group, WiAUC, WoAUC, and WiWoAUC were higher in subjects with Stage III CKD than in those with Stage I–II CKD. This result showed that CysC was normal in Stage CKD III patients, while renal blood perfusion parameters were larger than CKD I–II patients, we believe that renal microperfusion abnormalities may occur earlier than renal biomarker changes in CKD patients, but the number of CKD III patients (only 6 cases) was limited, so the results needed to be clarified by further increasing the number of patients. No previous studies have assessed the relationship between CEUS and CysC levels. We believe that CEUS parameters may help identify some patients with early-stage CKD misdiagnosed by CysC level.

We also explored the correlations between CysC level and microvascular perfusion assessed by CEUS. WiAUC, WoAUC, and WiWoAUC were selected because of their early performance in CEUS. The results showed that CysC was negatively correlated with WiAUC, WoAUC, and WiWoAUC. We also analyzed the relationship between eGFR, BUN, Scr, and the three CEUS parameters. The eGFR was positively correlated and BUN and Scr levels were negatively correlated with WiAUC, WoAUC, and WiWoAUC. Between the two methods of evaluating renal function, the correlation coefficient of CysC was higher than that of eGFR. Basic studies have shown that CysC might participate in diabetic microangiopathy by activating the neutrophil-mediated inflammatory response to cause endothelial dysfunction, or by interacting with homocysteine and cathepsin to promote the proliferation of vascular smooth muscle cells and damage to vascular endothelial cells [12,30]. At present, our clinical research results only observed the correlation between CysC and CEUS parameters. The mechanisms require further verification in more rigorous basic research.

Our study had some limitations. First, owing to the limited sample size and single-center design, additional studies with larger sample sizes and more medical centers are needed. Second, contrast medium was injected using the bolus method rather than an injection pump at a continuous rate. Although manual injection may reduce the accuracy of contrast injection, most studies still use this method.

## Conclusion

CEUS parameters reflected changes in renal microvascular perfusion in patients with DKD. Moreover, WiAUC, WoAUC, and WiWoAUC might be useful indicators of declining glomerular filtration rate in patients with DKD with increased CysC levels. However, these predictions warrant further validation. CEUS, as a noninvasive method, has potential clinical value for evaluating changes in renal function in patients with DKD.
